# Micro Droplet Formation towards Continuous Nanoparticles Synthesis

**DOI:** 10.3390/mi9050248

**Published:** 2018-05-18

**Authors:** Marek Wojnicki, Magdalena Luty-Błocho, Volker Hessel, Edit Csapó, Ditta Ungor, Krzysztof Fitzner

**Affiliations:** 1 Faculty of Non-Ferrous Metals, AGH University of Science and Technology, Al. A. Mickiewicza 30, 30-059 Krakow, Poland; mlb@agh.edu.pl (M.L.-B.); fitzner@agh.edu.pl (K.F.); 2 Micro Flow Chemistry and Process Technology, Department of Chemical Engineering and Chemistry, Technische Universiteit Eindhoven, P.O. Box 513, 5600 MB Eindhoven, The Nederland; v.hessel@tue.nl; 3 MTA-SZTE Biomimetic Systems Research Group, Department of Medical Chemistry, Faculty of Medicine, University of Szeged, Dóm tér 8., 6720 Szeged, Hungary; tidecs2000@yahoo.co.uk (E.C.); ungord@chem.u-szeged.hu (D.U.); 4 Department of Physical Chemistry and Materials Sciences, University of Szeged, Aradi Vt. 1., 6720 Szeged, Hungary

**Keywords:** sequential flow, droplets, microreactor, two-phase flow, simulations of 2D flow, COMSOL Multiphysics

## Abstract

In this paper, micro droplets are generated in a microfluidic focusing contactor and then they move sequentially in a free-flowing mode (no wall contact). For this purpose, two different micro-flow glass devices (hydrophobic and hydrophilic) were used. During the study, the influence of the flow rate of the water phase and the oil phase on the droplet size and size distribution was investigated. Moreover, the influence of the oil phase viscosity on the droplet size was analyzed. It was found that the size and size distribution of the droplets can be controlled simply by the aqueous phase flow rate. Additionally, 2D simulations to determine the droplet size were performed and compared with the experiment.

## 1. Introduction

Nowadays, continuous-flow systems are widely used for chemical synthesis and flow chemistry has become a versatile approach in modern synthetic chemistry [1]. There are manifold benefits of using flow chemistry and the most prominent one is improvement of yield [2] or selectivity by more controlled processing, e.g., in terms of mixing (mass transfer) [3] and/or heat transfer. Besides, that activation is provided by the use of high temperature [4], solvent-free processing [5], photo-irradiation [6], and others. This has been termed the ‘novel process window’ [7]. This allows for low reagents consumption and produces a small amount of waste. Also, for some low scale applications, the micro flow systems are more economical as compared with conventional processing [8,9,10].

The top 30 petrochemicals and most of the top 300 organic chemicals are manufactured on a large scale using dedicated, continuous processing plants which run without shutdown for many years. Batch systems have always been preferred on a small scale, because of their simplicity in use and versatility. Yet, continuous processing on a small scale has, in the last decade, got into industrial and academic foci. This is due to its function enabling of the intensification of the synthesis processes and the quest for chemical plant modularization and automation [11].

As a result, the Food and Drug Administration (FDA) has called upon pharmaceutical manufacturers to switch from batch to continuous processing by the year 2026 and consequently, flow chemistry slowly becomes standard in pharmaceutical processing [12,13,14].

Among the variety of small-scale continuous-flow apparatus, micro-flow systems (‘microreactors’) [15] are the spearhead of process intensification innovation, and the chemical process technique called ‘flow chemistry’ [16,17,18,19] aimed at pharmaceutical synthesis is their major application. A simple and even inherent advantage of these micro-flow systems is related to their small inner volumes, which also means small volumes of processed chemicals used during experimental synthesis [17,20].

There is a large number of reports showing potential application of micro flow systems for continuous nanoparticles synthesis [6,21,22,23,24], composite materials [25,26,27,28,29,30], as well as for metal ion extraction and separation [31].

Continuous, nanoparticle synthesis does not only allow intensification of the process, but it can also control particle size, size distribution, and shape [32,33,34]. In the case of shape control, it seems that the micro flow systems open up new possibilities. Robertson in his review paper has shown a significant number of examples where continuous flow systems are used to direct synthesis and assembly of nanomaterials and nanocomposites [35]. It seems that there is no willingness to move from the round-bottomed flask to the microflow system, despite obvious advantages [36].

In this particular case the main benefit of the micro flow system is to enable very fast mixing and phase contacting much beyond the standards of conventional micromixers or contactors, respectively. In the case of nanomaterial formation, the micro flow has strong influence on the nanoparticle size distribution. It is worth noting that passive micro mixing typically takes place, which uses the pumping energy, possibly superimposed by flow obstacles such as zig-zag, helical, split-recombine, staggered herringbone, and more geometries [37]. The best defined conditions are given in diffusion-only micro mixers which have high flow symmetry and can be thus best accessed by simulation. Active micro mixers—i.e., making use of electrical, magnetic or ultrasound energy at typically low pumping energy—are another choice; however, such mixing systems commonly have longer mixing times than passive ones and are much more complicated to be fabricated. Therefore, it is not surprising that there are only few reports on active macromixing topic [38,39,40].

Another method that allows for good mixing by virtue of convection in the nanoparticle synthesis is the application of micro droplets. The idea of using micro droplets for various applications has been a hot topic for a decade, and it is evidenced by a large number of publications which appeared on this subject [41]. This type of droplet microfluidics is applied in biomedicine [42] and mostly for all kinds of flow chemistries. Droplet microfluidics and segmented flow have also been used for nanomaterial synthesis [43,44]. After synthesis, the organic phase can be separated from the aqueous system using membrane. Also, this process can be conducted using a continuous flow system [45]. It should be noted, that in the case of microdroplets, their separation by gravity force is not very efficient.

**Figure 1:**
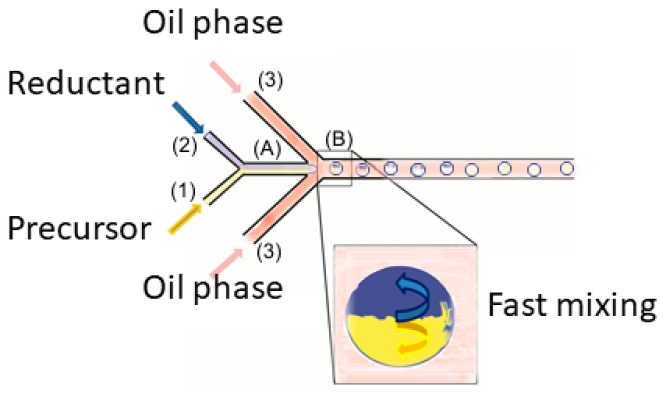
Droplet microfluidics system inducing inside droplet convective mixing of a formerly bilayered reaction system.

The concept of metal nanoparticle synthesis inside a droplet or slug which is formed at the interface of the water and oil phase and induces convective mixing, is shown Figure 1. Ahead of this phase contactor junction, another junction is given to mix a metal precursor and reductant solution to give one phase. In the next stage, the water phase is surrounded by the oil phase and a droplet is formed. Inside the droplet, fast mixing sets in [41,46,47] and the reaction between metal precursor and reductant takes place. The mixing maintains over the whole fluidic path long. Additionally, the ‘droplet train’ can be considered as a series of moving batches which all have the same residence time, i.e., residence time distribution is very narrow and axial back mixing is practically absent. Moreover, in the case of continuous micro flow system application, adsorption of nanoparticles on the walls was observed and described in the literature [26,48]. This effect may lead to micro reactor blocking. To prevent this effect, microdroplets seem to be promising tools for colloid continuous synthesis.

In the case of microreactor shown in Figure 1, a part of the reactor may be exposed to contact with reacting compounds. This in turn will inevitably induce fouling. The problem in fact, is directly related to the rate of the reaction. If the reaction is fast, the deposition may take place in this area. However, if the reducing agent is not strong, the effective reducing time is longer then resident time in nozzle range. In case of this system, the resident time is ca. 0.005 s when the flow rate is equal to 2 mL/h.

The dimensions of the droplet are limited on the one hand by the channel size, and on the other hand it can be influenced by the flow conditions (flow rates of the particular phases and flow rate ratio).

The potential application of microreactors and microdroplets for liquid–liqiud extraction is extremely interesting. There are several reports where microreactors were used for metals separations e.g., Co(II)-Ni(II) [31,49], and extraction U(VI) [50], Eu(III) [51], Zn(II) [52], Cu(II) [53] in flow systems. Thanks to that, increase in selectivity and process intensification was obtained.

In this paper, we would like to compare the slug size obtained from a mathematical model with the results of the experiment. For this purpose, 2D simulations were performed.

## 2. Experimental

In the experiments three different microfluidic devices were used, all delivered by Dolomite Microfluidics (Royston, UK) (for details, see Supplemental Figures S1–S3).

The aqueous phase was stained using methylene blue (delivered by Avantor Performance Materials, Center Valley, PA, USA), which is insoluble in the oil phase. The mixture of heptane (Avantor Performance Materials) and methyl silicone Polsil OM 10 (purchased from Zakład Chemiczny Silikony Polskie, Nowa Sarzyna, Poland) in different ratio *v*/*v*, were used as the oil phase. As the droplets stabilizers, Span 80 (delivered by Sigma Aldrich, St. Louis, MO, USA), PVA (Sigma Aldrich, MW = 67,000), and Tween 80 (Avantor Performance Materials) were used. The last two polymers (PVA and Tween 80) may also act as silver nanoparticle stabilizing agents.

In all experiments, water phase contained 1% *m*/*v* of PVA and 1% *v*/*v* Tween 80. In the case of hydrophobic microfluidic device, the oil phase was also enriched with the addition of 1% *v*/*v* of Span 80.

To obtain low flow pulsation during experiments, syringe pumps (Injectomat Agilia, Fresenius Kabi AG, Homburg, Germany), were applied. In the experiments, syringes of 60 mL capacity were used. It has to be noted, that the piston seal in a typical medical syringe is not resistant to heptane, and therefore they must be replaced frequently (ca. 3 h).

The nozzle size is small so, to prevent its occlusion, all reagents were filtered using 200 nm syringe filters. The filters were directly mounted on the syringe placed in the pump. All joints and capillaries were made of Teflon. Droplets formation process is sensitive to external disorders, like vibrations and shocks. After setting new experimental conditions (flow rates changes), ca. 5–10 min is required to obtain a stable droplets formation process.

Observations of droplets formation were performed using microscope Optek SZM7045T-STL2 (Optek Technology, Woking, UK), equipped with a digital camera Sony α N5, and/or FPS1000 (Sony Corporation, Tokyo, Japan). For the registered image analysis and the droplet size and size distribution determination, the ImageJ software ver. 1.45s (National Institutes of Health (NIH), Bethesda, MD, USA) was used.

The Höppler viscometer (Thermo Scientific™ HAAKE™ Falling Ball Viscometer C, Waltham, MA, USA) was used to determine the oil phase viscosity. All experiments were performed at room temperature (296 K).

The simulation of droplets formation was carried out using COMSOL Multiphysics 4.3 (COMSOL, Inc., Burlington, MA, USA).

The contact angles of water at the surface of micro reactors were determined experimentally and are equal to 2.3 (rad) and 0.38 (rad) for hydrophilic and hydrophobic reactors, respectively.

## 3. Hydrophilic Chip Application

**Figure 2:**
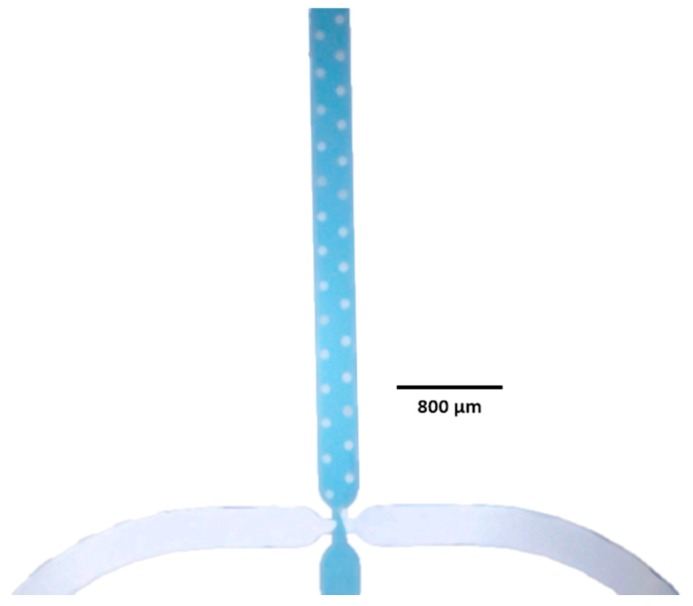
Microscopic view of oil droplet formation in the water phase. The aqueous phase is stained blue. *V*_oil,T_ = 0.2 mL/h and *V*_H_2_O,T_ = 5 mL/h and hydrophilic surface.

The surface properties of the glass microfluidic devices are important factors during the droplet synthesis [54,55,56]. In the case of hydrophilic glass, the surface is covered with hydroxyl groups. Those groups interact with water molecules by weak hydrogen bonds. In effect, water molecules stack to the surface in the form of a thin layer. Therefore, in this type of microreactors water droplets are difficult to form. Consequently, a mixture of an aqueous phase with an oil phase generates only oil droplets, as shown in Figure 2. This configuration might also have important applications in the synthesis of hydrophobic nanoparticles [57,58]. In general, the synthesis of hydrophobic nanoparticles can be performed either in the aqueous system, functionalized in the next step to obtain hydrophobic nature [59], or directly in the organic phase [60].

**Figure 3:**
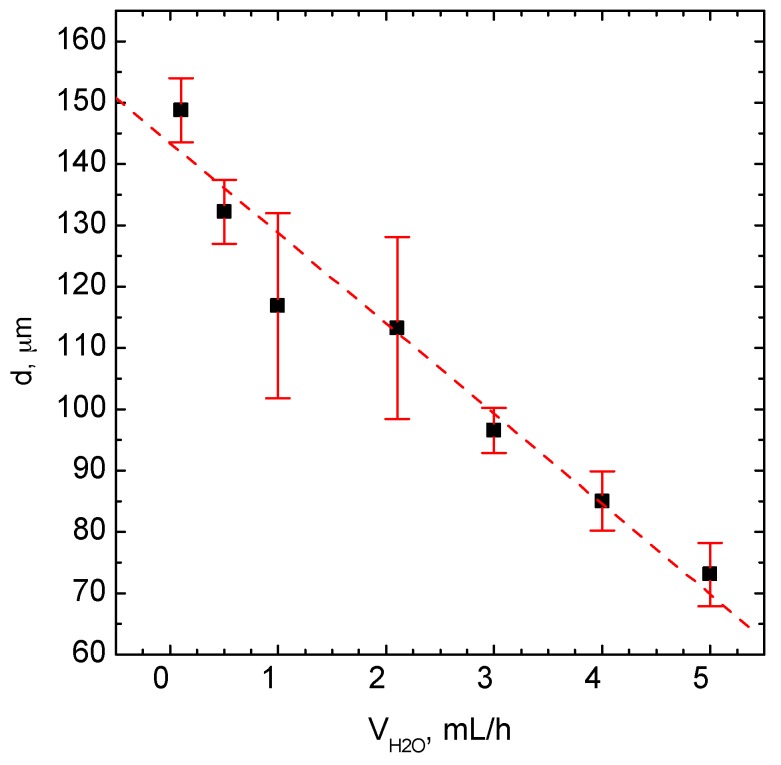
The influence of the water flow rate on droplet size. Conditions: *V*_oil_ = 1.5 mL/h.

For all experiments with the hydrophilic glass chip, Polsil OM 10 oil was used. In Figure 3, the influence of water phase flow rate on the droplet size and size distribution is shown. The oil phase flow rate was kept constant and was equal to 0.1 mL/h per input channel.

It can be seen that an increase of water flow rate reduces the droplet size from 150 µm to 75 µm (diameter). This dependence seems to be linear in the studied range of flow rates.

**Figure 4:**
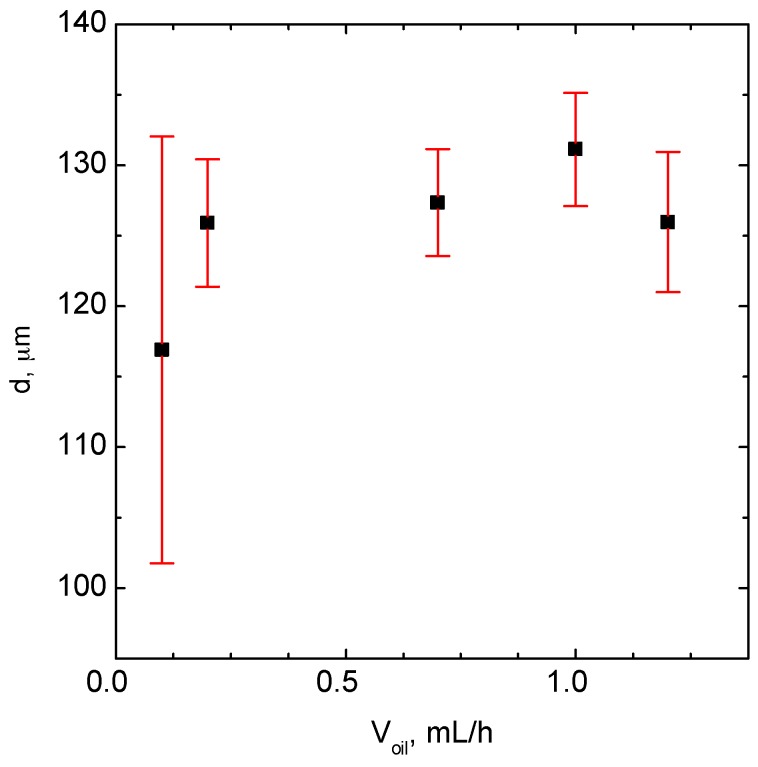
The influence of the oil phase flow rate on droplet size and size distribution. Conditions: *V*_H_2_O_ = 1 mL/h.

Next, the influence of the oil phase flow rate on the droplet size and size distribution was also investigated. The obtained results are shown in Figure 4. It is demonstrated that there is no significant effect of the oil flow rate on the droplets’ size and size distribution.

In this experiment, the flow rate of the water phase was kept constant and was equal to 1 mL/h.

## 4. Droplet Formation in Hydrophobic Chip

**Figure 5:**
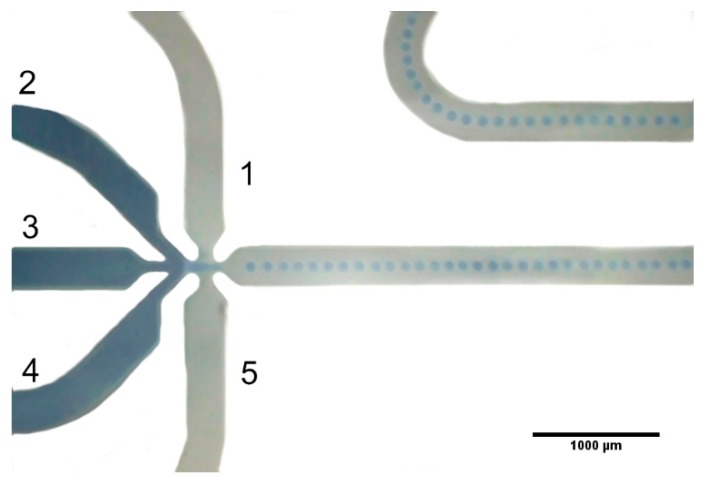
View of the water droplet formation in the micro channel arrangement.

In the case of hydrophobic chip application, the micro channel arrangement as well as a typical droplet formation are shown in Figure 5.

The micro reactor consists of 5 input channels. Channels 1 and 5 were used to deliver the oil phase. Those channels are connected with one input (not shown in the photo). Thanks to that, only one pump is required to feed the system.

Channels 2 and 4 are connected in a similar way to channels 1 and 5. Channel 3 is single and requires an additional third pump. The channel diameter is equal to 300 µm. In the nozzle region its diameter is decreased down to 100 µm. Channel length after junction 197mm. Volume of channel after junction equals 5.9 µL.

**Figure 6:**
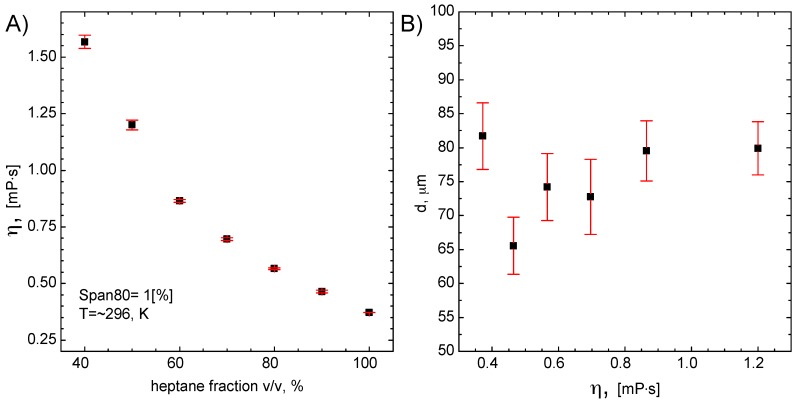
The viscosity of the oil phase vs heptane fraction (**A**), and the droplet size and size distribution vs. viscosity (**B**). Experimental conditions: total water flow rate 1 mL/h, total oil flow rate 5 mL/h.

In Figure 6B, the influence of oil phase viscosity on the droplet size and size distribution is shown.

The oil phase viscosity was changed by mixing the heptane with the Polsil OM10 with a different volumetric ratio. The viscosity of pure Polsil OM10 is equal to 10 mP·s. Due to the mixing of heptane with the increasing amount of Polsil OM10, the viscosity of the system also increases (Figure 6A).

It can be seen that there is no significant influence of the oil phase viscosity on the droplet size and size distribution (Figure 6B). This, in turn, suggests that other parameters may have an impact on the droplet size.

**Figure 7:**
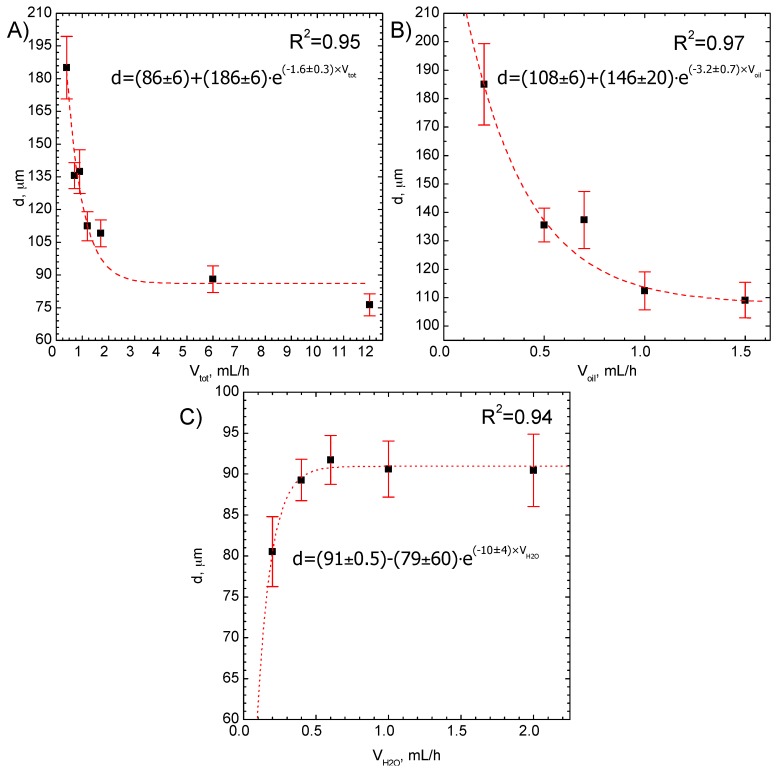
The influence of the total flow rate (**A**), oil phase flow rate when the water flow rate is constant and equal to 1 mL/h (**B**) and water phase flow rate when the oil flow rate is constant and equal to 5 mL/h (**C**) on the droplet size and size distribution.

As it was mentioned in the experimental part that the droplet size was determined using imageJ software. However, we did not make it automatically. The reason for large error bars is directly related to the low contrast of the droplets’ edges. Lan [61] et al. have also investigated the influence of viscosity on the droplet size. They observed only limited influence of this parameter. However, in their experiment, the geometry of the system is significantly different. Li [62] et al. have described the effect of viscosity onto droplet size. They have found that the size of the droplets can be controlled by changes of oil phase viscosity. In the described case, oil phase viscosity varied in the range from 21 mm^2^/s to 194.6 mm^2^/s. By changing the viscosity of the oil phase about ten-fold, it was possible to change the droplets diameter from 80 µm to 120 µm only. It has to be noted that viscosity value was 100 times larger than in our case. Therefore, a deeper analysis of the flow parameters on the droplet size is needed. The obtained results of those experiments are shown in Figure 7.

The influence of the flow rate of oil, water, as well as the total flow rate on the droplet size and size distribution is shown in Figure 7A–C. This influence can be described by an exponential function

(1) $$d=d_{\min}+Ae^{-kV_{x}}$$

where: *d* and *d*_min_ denote diameter and minimal diameter of a droplet, respectively; *A*, *k* are constant coefficients; *V_x_* denotes *V*_tot_—total flow rate, *V*_oil_—oil flow rate, and *V*_H_2_O_—water flow rate, respectively.

In each case, the droplet size tends to reach a constant value (*d*_min_), which is independent of the flow rate. It also confirms, that the achievable minimum value is about 95 µm and can be correlated with the size of the nozzle region in the microreactor, where the drop formation begins (Figure 5).

A strong effect on droplet size is related to the change in oil phase flow rate (Figure 7B). It was shown that the droplet size can be reduced from 185 ± 15 µm for the smallest flow rate of oil phase (0.2 mL/h) to 110 ± 10 µm for the highest flow rate of oil phase (1.5 mL/h). In the case of the aqueous phase (Figure 7C), an increase of the flow rate causes an increase of the formed droplet size from 80 ± 5 µm up to 90 ± 8 µm.

## 5. Simulation of Droplet Formation

Simulation of droplet formation was performed using COMSOL Multiphysics, taking into account the two phase flow level set interface model. From the modeling point of view, the droplet formation can be described as

(2) $$\rho \frac{\delta u}{\delta u}+\rho \cdot (u\cdot \nabla )u=\nabla \left[-pI+\mu \left(\nabla u+{\left(\nabla u\right)}^{T}\right)\right]+F_{\mathrm{st}}$$

(3) ∇⋅u=0

(4) $$\frac{\delta \phi }{\delta t}+u\cdot \nabla \phi =\beta \left(-\phi (1-\phi \right)\frac{\nabla \phi }{\left|\nabla \phi \right|}+\varepsilon \nabla \phi )$$

where: ϕ is the level set function and the β and ε are numerical stabilization parameters, *u* is the velocity (m/s), *t* is the time (s), *µ* is the dynamic viscosity (Pa·s) and the *p* is the pressure (Pa). The *F*_st_ is the surface tension force (N/m) and can be approximated using Antonoff’s [63] rule as

(5) $$F_{\mathrm{st}}=\gamma_{H_{2}O}^{\mathrm{air}}-\gamma_{\mathrm{oil}}^{\mathrm{air}}$$

where $\gamma_{H_{2}O}^{\mathrm{air}}$ is the surface tension of water and $\gamma_{\mathrm{oil}}^{\mathrm{air}}$ is the surface tension of oil phase measured in the contact with air.

There is general agreement that this model describes this physical phenomenon well [64,65]. Simulations were performed assuming experimental conditions and reactor geometry as described in Section 4.

**Figure 8:**
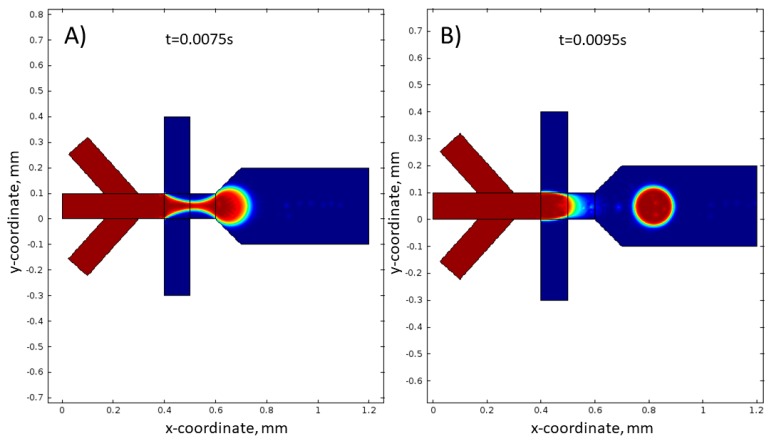
Simulation of droplet formation in the hydrophobic micro reactor. (**A**) After 0.0075 s and (**B**) after 0.0095 s from the beginning of the experiment. Experimental conditions: *V*_H_2_O,T_ = 1 mL/h, *V*_oil,T_ = 2 mL/h.

Simulations were performed for the following experimental conditions: total flow rate of water phase *V*_H_2_O,T_ = 3 mL/h, and total flow rate of oil phase *V*_oil,T_ = 4 mL/h. It was assumed that, for each inlet, the flow rate is equal one-third *V*_H_2_O,T_ and one-half *V*_oil,T_. The obtained results are shown in Figure 8.

The presented simulations show details of the droplet formation, i.e., creating the characteristic hydrodynamic focusing (Figure 8A) and droplet break-off (Figure 8B) in time. The size of the droplets determined by mathematical model is equal to 105 µm. This value is about 15 µm bigger than droplet size determined in the experiment (Figure 7A). This difference may result from measurement of droplet diameter uncertainty during image analysis. It has to be noted that the contrast of analyzed droplets is low, especially on their edges.

**Figure 9:**
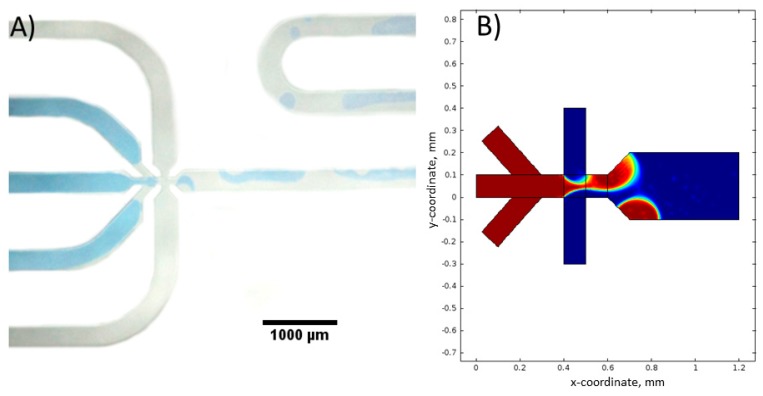
(**A**) Formation of droplets in the hydrophilic micro reactor and (**B**) simulation of a droplet formation in the hydrophilic reactor, experimental conditions: *V*_H_2_O,T_ = 1 mL/h, *V*_oil,T_ = 3 mL/h.

The 2D simulation of the droplet formation process in the hydrophilic reactor was also performed. An example of each of the experimental results and the simulations is shown in Figure 9A,B, respectively.

In the hydrophilic reactor, no flow of water droplets is observed (Figure 9A). The formed water bubbles are only sliding along the walls of the reactor. The simulation performed for the same condition confirmed the same fluid behavior (Figure 9B). It can be concluded that the chemical properties of the reactor surface play an important role in the process of oil and water droplets formation.

## 6. Conclusions

Using a simple system consisting of oil and water phase, it is possible to form stable microdroplets with narrow size distribution. The droplet size and size distribution can be controlled easily by the change of the flow rate of the oil phase. The chemical properties of the reactor walls have significant impact on the droplet formation. The obtained results show that the produced droplets with uniform morphology are a promising tool for metal nanoparticle synthesis or liquid–liquid extraction. Moreover, it was shown that the assumed mathematical model describes the process of droplet formation in a microreactor well. Thanks to that, it is possible to reduce the number of expensive experiments conducted in a two phase micro reactor.

## Supplementary Materials

The following are available online at http://www.mdpi.com/2072-666X/9/5/248/s1, Figure S1: The microsystem for microdroplets formation and analysis; Figure S2: The hydrophilic/hydrophobic chip; Figure S3: Droplet junction chip.
