# Supplementary material for: Micro Droplet Formation towards Continuous Nanoparticles Synthesis
Source: Micromachines (Basel). 2018 May 18;9(5):248. doi: 10.3390/mi9050248 (PMC6187485; doi:10.3390/mi9050248)
Supplement: Supplementary file 1 [file micromachines-09-00248-s001.pdf]

Supplementary Materials: Micro Droplet Formation towards Continuous Nanoparticles Synthesis

Marek Wojnicki, Magdalena Luty-Błocho, Volker Hessel, Edit Csapó, Ditta Ungor and Krzysztof Fitzner

Microfluidic Devices for Microdroplets Formation.

The process of microdroplets formation were carried out in microsystem shown in Figure S1. The set up consist of two pumps (A, B), glass chip (D), microscope with digital camera (C) and sample collector (G).

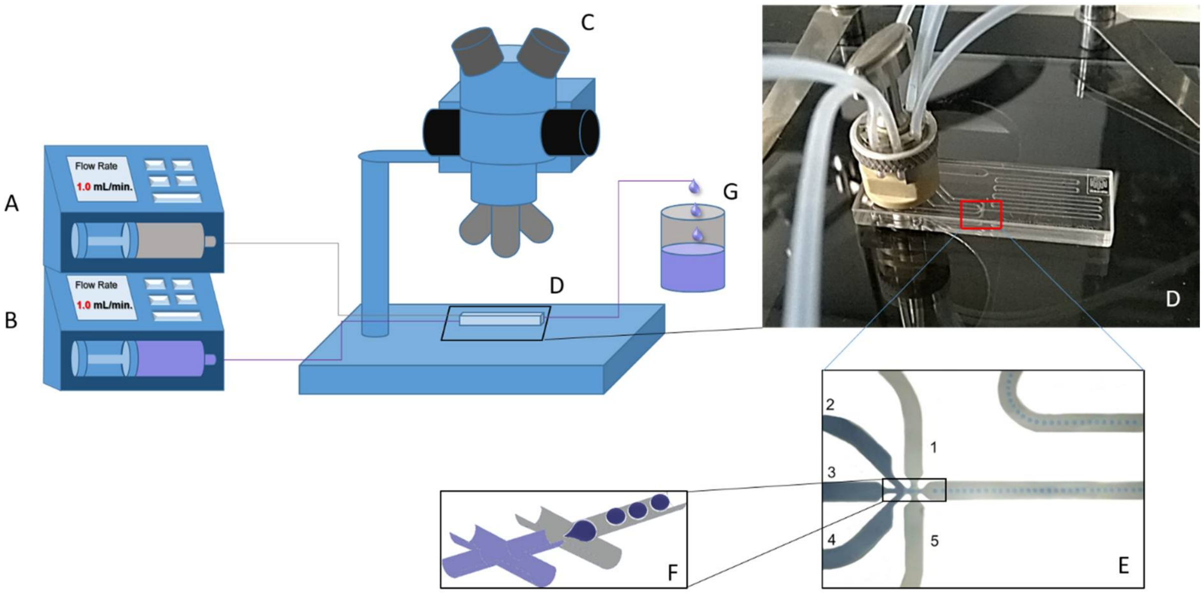

Figure S1. The microsystem for microdroplets formation and analysis. (**A**) Pumps with water phase; (**B**) Pumps with oil phase; (**C**) Microscope with digital camera; (**D**) Chip; (**E**,**F**) Details of microdroplets formation; (**G**) Sample collection; 2, 3, 4—water phase inputs; 1, 5—oil phase inputs.

Figure S2 shows the hydrophilic/hydrophobic chip for microdroplets formation.

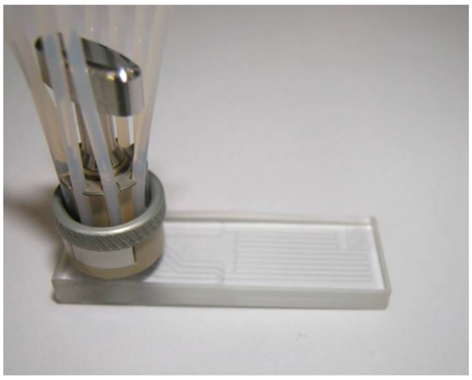

Figure S2. The hydrophilic/hydrophobic chip.

Figure S2 shows droplet generation chip with header, which is a glass microfluidic device designed for generating droplets made up of one, two or three reagents. This chip is fabricated in process of HF etching and thermal bonding. Standard Chip 100 μm channel depth has 4 inputs and 2 outputs. Channel cross-section at junction equals 100 μm (depth) and 105 μm (width). Channel

length after junction 197 mm. Volume of channel after junction equals 5.9 µL. Operating pressure 20 Bar. More details can be found in product datasheet [1].

Figure S3 shows the hydrophilic chip (droplet junction chip) for microdroplets formation.

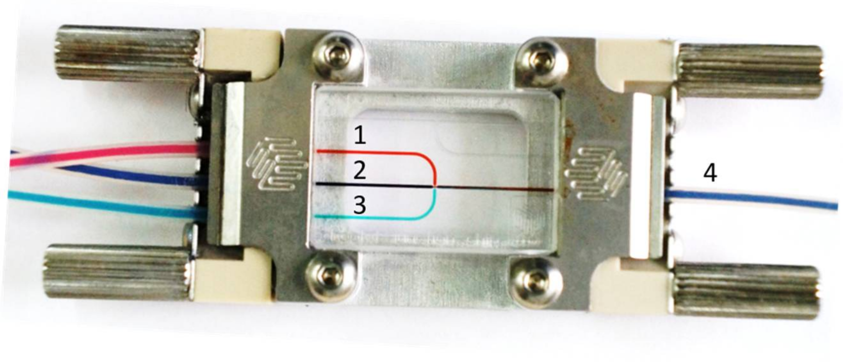

Figure S3. Droplet Junction Chip. 1–3 denote inputs; 4—output.

This chip is made of glass and fabricated in process of HF etching and thermal bonding. In experiments X-junction channel format was used as shown in Figure S3. Droplet Junction Chip 100 µm etch depth has 3 inputs and 1 output. Channel cross-section at junction equals 100 µm (depth) and 105 µm (width). Channel length after junction 11.25 mm. Volume of channel after junction equals 0.31 µL. Operating pressure 30 Bar. More details can be found in product datasheet [2].

Reference

1. Droplet Generation Chip with Header. Available online: http://marketing.blacktrace.com/acton/attachment/28584/f-02c5/1/-/-/-//Droplet%20Generation%20Chips%20with%20Header.pdf (accessed on 21 July 2017).
2. Droplet Junction Chips. Available online: http://marketing.blacktrace.com/acton/attachment/28584/f-02c6/1/-/-/-/-/Droplet%20Junction%20Chips.pdf (accessed on 21 July 2017).
